# *Shigella* humoral immunity during the first 2 years of life in children from endemic areas

**DOI:** 10.1128/mbio.00555-25

**Published:** 2025-04-16

**Authors:** Esther Ndungo, Ushasi Bhaumik, Yuanyuan Liang, Wilbur H. Chen, Mark A. Travassos, Milagritos D. Tapia, Karen L. Kotloff, Myron M. Levine, Marcela F. Pasetti

**Affiliations:** 1 Center for Vaccine Development and Global Health, University of Maryland School of Medicine 12264, Baltimore, Maryland, USA; 2 Department of Epidemiology and Public Health, University of Maryland School of Medicine, Baltimore, Maryland, USA; Fondazione Biotecnopolo di Siena, Siena, Italy

**Keywords:** *Shigella*, multiplex assay, antibodies, seroprevalence, children

## Abstract

*Shigella* is a leading cause of moderate-to-severe diarrhea, primarily affecting children in impoverished regions. Disease incidence is highest in toddlers 1–2 years of age. Acquisition of immunity over time reduces the risk of infection. However, the evolution of childhood immunity resulting from *Shigella* exposure and the host responses that mediate protection remain poorly understood. Gaining insight into the immunological features that develop over time and prevent subsequent *Shigella* infection is critical for guiding vaccine design. We examined the antigenic repertoire and magnitude of humoral immunity to *Shigella* in children < 2 years of age from three endemic areas—Bamako, Mali, and two sites (Afar and Tigray) in Ethiopia—using a qualified multiplex assay. Serum IgG and IgA specific to *Shigella* proteins (IpaB, IpaC, IpaD, IpaH, and VirG) and lipopolysaccharide (LPS) from *S. flexneri* 2a, *S. flexneri* 3a, *S. flexneri* 6, and *S. sonnei* were measured in three stratified age groups: 6–8, 12–17, and 18–24 months. The youngest group (6- to 8-month-olds) exhibited similar antibody profiles across all sites. By contrast, antibody levels in the 12- to 17- and 18- to 24-month-old age groups varied by both age and site, reflecting geographical differences in *Shigella* endemicity and exposure. Notably, most children in the 12- to 17-month-old age group had IgG levels below the adult protective thresholds identified in controlled human infection models, identifying a critical window of vulnerability that would require intervention. Our findings underscore the importance and value of *Shigella* serosurveillance in children from endemic regions to inform future vaccine rollout decisions.

**IMPORTANCE:**

*Shigella* is a major cause of moderate-to-severe diarrhea, the most affected being young children from poor resource countries. *Shigella* species easily acquire antibiotic resistance, presenting a challenge to infection control. The development of vaccines for young children has been hindered by a lack of understanding of what constitutes protective immunity. Here, for the first time, we investigated the magnitude and specificity of *Shigella* humoral immunity evoked by natural exposure in children 6 months to 2 years old living in Mali and Ethiopia (*Shigella*-endemic areas) using a qualified multiplex assay. Antibody profiles varied with age and region, revealing epidemiological trends. Children 12–17 months old were identified as the most vulnerable to infection. Antibodies specific for conserved *Shigella* proteins were higher in older children, affirming their potential as vaccine candidates. *Shigella* serosurveillance is useful in guiding public health interventions.

## INTRODUCTION

*Shigella* spp. continue to account for more than 60,000 deaths yearly in children under 5 years of age globally (1). Recurrent infection can lead to debilitating sequelae including malnutrition, stunted growth, and deficits in immune and cognitive development (2–4). A safe, effective, and affordable *Shigella* vaccine targeting this age group in low-resource settings would make a major public health impact (5, 6).

The pattern of risk of *Shigella* infection during the first 5 years of life follows a bell-shaped curve: infants 0–11 months old have a low risk of infection (7–9); incidence of disease peaks at 12–23 months of age and then declines in children 24–59 months of age. Meanwhile, *Shigella* immunity follows the exact opposite trend: infants have high levels of maternally derived antibodies against *Shigella* at birth (10, 11), which gradually decline during the first year of life (10, 12) creating a window of vulnerability that coincides with the highest incidence rates. *Shigella* immunity accrues thereafter in older children, after exposure, resulting in an age-dependent gain in protective immunity. Understanding the breadth and progressive development of immunity to *Shigella* during childhood, which confers protective status to older age groups in endemic areas, is important to identify effective immune signatures and relevant vaccine target antigens. An interrogation of the antibody landscape can reveal epidemiological trends and host defense elements that contribute to protection and guide vaccine development efforts.

There is a paucity of knowledge on the evolution of *Shigella* immunity in young children. The studies that exist have focused on the *Shigella* lipopolysaccharide (LPS) (10, 12–18) and specific serotypes. Serotype-specific immunity directed to the O-specific polysaccharide acquired through *Shigella* infection is a putative correlate of protection (19, 20). By contrast, the analysis of antibodies to conserved *Shigella* proteins has lagged; however, they have been deemed important based on the antigenic repertoire observed in adults from endemic areas who acquire natural immunity (11) and protective signatures identified in controlled human infection model (CHIM) studies (21, 22). The extent to which children mount immunity to bacterial antigens/virulence factors other than LPS and the nature of these responses remain poorly understood.

In this study, we report the development of a multiplex immunoassay using the Meso Scale Discovery (MSD) platform that enables the simultaneous quantification of 9 *Shigella* antigens: invasion proteins (Ipa) B, IpaC, IpaD, and IpaH, the virulence protein VirG, and LPS antigens representing the most prominent circulating serotypes: *S. flexneri* 2a, *S. flexneri* 3a, *S. flexneri* 6, and *S. sonnei* (23, 24). This assay was subjected to a full analytical qualification to ensure appropriateness for the intended use and reliability. Seeking to understand the acquisition of immunity in young children living in endemic regions, we used this platform to conduct a cross-sectional analysis of the *Shigella* antibody repertoire in infants 6–8 months of age and in toddlers 12–17 and 18–24 months of age living in Mali and Ethiopia. Our analysis revealed epidemiological trends and age-specific vulnerability against shigellosis in these populations.

## RESULTS

### Assay qualification

#### Optimization of assay conditions

A multiplex assay for simultaneous detection of *Shigella* antibodies of diverse specificities was developed using the Meso Scale Discovery (MSD) technology. The target antigens included LPS from common circulating *Shigella* serotypes detected in children living in Sub-Saharan Africa (8, 23, 24): *S. flexneri* 2a, *S. flexneri* 3a, *S. flexneri* 6, and *S. sonnei*, as well as *Shigella* type III secretion (T3SS) proteins IpaB, IpaC, IpaD, IpaH, and the virulence protein VirG. Plates were custom printed by MSD using optimal antigen coating concentrations (i.e., highest ECL signal-to-noise ratios) identified during initial testing. Assay reagents (diluents, washing and blocking buffer, incubation, and secondary antibodies) and conditions (incubation times and temperature) were also selected to increase specific signal and dynamic range.

#### In-house standard curves

**Fig 1:**
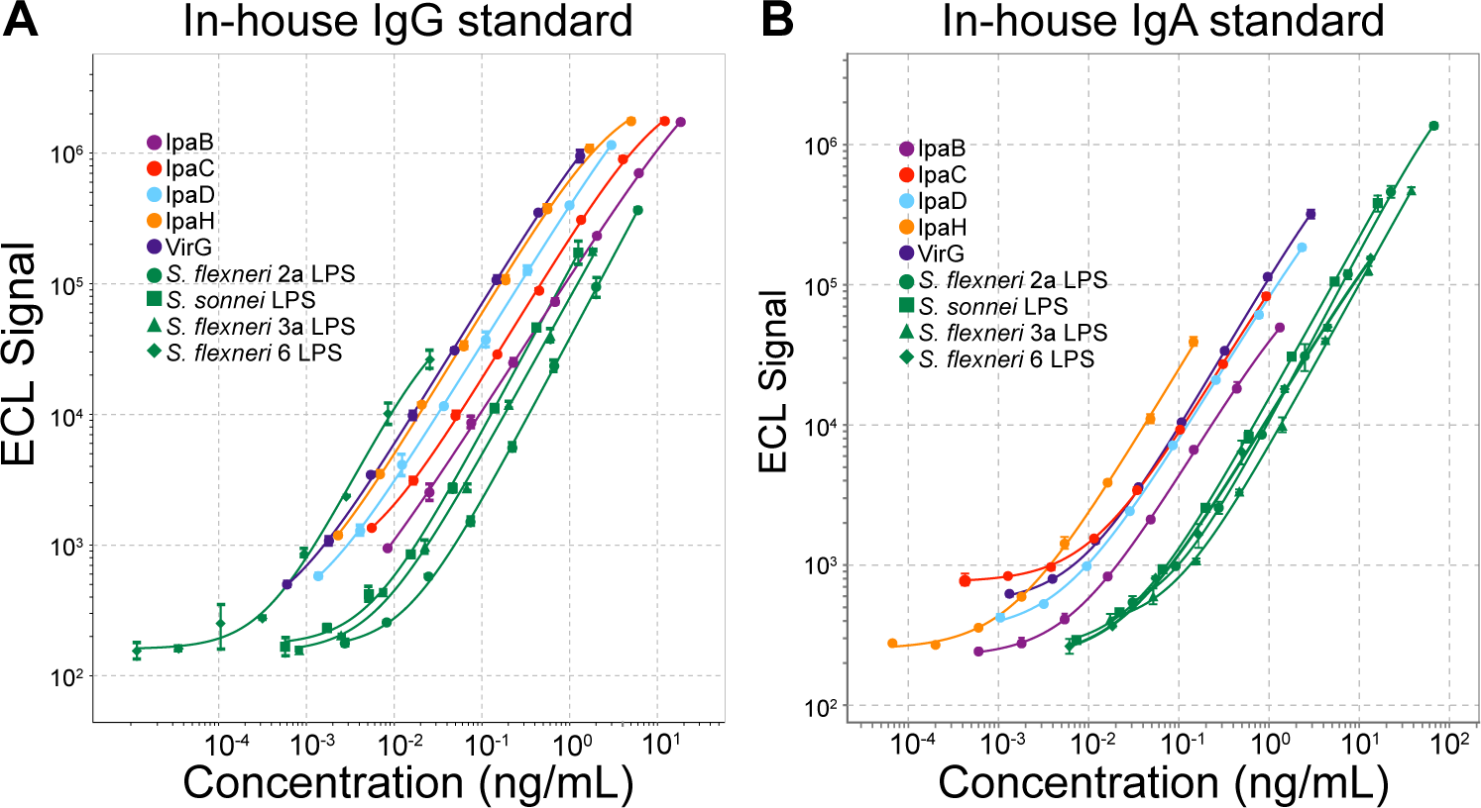
In-house standard dose-response curves for all nine antibody specificities generated by the *Shigella* multiplex immune assay. Representative curves of ECL values (signal) vs antibody concentration (ng/mL) are shown spanning eight threefold serial dilutions of the in-house standards at the selected starting dilutions: 1:10,000 for IgG (**A**) and 1:100 for IgA (**B**). The antibody concentration for each antigen (ng/mL) had been pre-assigned using the Zollinger method.

Efforts to develop an international standard for *Shigella* serology are underway (25). Meanwhile, we established our own IgG and IgA in-house standards for use in various immunoassays (described in detail in Materials and Methods). The IgG in-house standard consisted of a pool of high titer sera from adult individuals living in an endemic area. The IgA in-house standard was a pool of breast milk from lactating women also from an endemic area (breast milk was used due to the low levels of IgA against some antigens in adult sera). IgG and IgA concentrations (ng/mL) were assigned based on a modified Zollinger method (26). Dose-response curves for each of these preparations are shown in Fig. 1A and B. Standard curve parameters (*R*^2^, hillslope, and detection limits) for each antigen are shown in Table 1.

**TABLE 1:** Standard curve parameters

Parameter	IpaB	IpaC	IpaD	IpaH	VirG	*S. flexneri* 2a LPS	*S. sonnei* LPS	*S. flexneri* 3a LPS	*S. flexneri* 6 LPS
In-house IgG standard									
Assigned concentration (ng/mL)	184,038	121,439	29,842	50,173	13,158	59,922	12,536	18,151	256
Coefficient of determination (*R*^2^)	0.999	0.998	0.999	0.991	0.999	0.999	0.999	0.998	0.998
Calculated hill slope	1.04	1.11	1.08	1.11	1.09	1.26	1.25	1.21	1.30
Detection limits (ng/mL)									
Calculated high	18.404	12.144	2.984	5.017	1.316	5.992	1.254	1.815	0.026
Calculated low	0.009	0.006	0.002	0.002	0.0007	0.005	0.003	0.001	0.0002
In-house IgA standard									
Assigned concentration (ng/mL)	132	93	232	15	289	6,722	1,601	3,787	1,338
Coefficient of determination (*R*^2^)	0.999	0.999	0.999	0.998	0.999	0.999	0.999	0.999	0.999
Calculated line slope	1.07	1.08	1.05	1.07	1.11	1.23	1.16	1.17	1.07
Detection limits (ng/mL)									
Calculated high	1.316	0.929	2.316	0.146	2.895	67.218	16.012	37.868	13.381
Calculated low	0.001	0.004	0.002	0.0001	0.002	0.052	0.011	0.034	0.015

Both the serum and breast milk standards produced linear curves with broad dynamic ranges. The IgG in-house standard curve showed a linear detection range of >3 logs for all antigens, except for *S. sonnei* LPS and *S. flexneri* 6 LPS (between 2 and 3 logs) (Fig. 1A and Table 1). The linear detection range for the IgA in-house standard also spanned >3 logs for all antigens with the exception of IpaC (Fig. 1B and Table 1). Despite the differences in antibody content, linear curves were achieved for all antibody specificities with a single dilution scheme for each standard. The assigned unitage of both standards is indicated in Table 1.

#### Limits of quantitation

The lower and upper limits of detection (LLOD and ULOD, respectively) for each antibody specificity were determined from the individual standard curves (Tables 2 and 3). The lower limits of quantification (LLOQ) were calculated by multiplying the LLOD by the minimum required dilution (MRD) which was set at 100 for this particular study to maximize ECL values falling within the acceptable detectable range (Tables 2 and 3). A ULOQ was not set; samples with ECL signals above the ULOD were re-tested at a higher dilution.

**TABLE 2:** IgG assay qualification parameters

Parameter	IpaB	IpaC	IpaD	IpaH	VirG	*S. flexneri* 2a LPS	*S. sonnei* LPS	*S. flexneri* 3a LPS	*S. flexneri* 6 LPS
Limits of quantitation									
LLOD (ECL signal)	9.41	6.27	1.66	2.50	0.76	6.23	2.41	3.97	0.09
LLOQ (ng/mL)	0.941	0.627	0.166	0.250	0.076	0.623	0.241	0.397	0.009
Linearity									
Slope	1.07	1.11	1.08	1.08	1.06	1.06	1.04	1.11	1.04
Coefficient of determination (*R*^2^)	0.999	0.999	0.999	1.000	0.999	0.994	0.998	0.991	0.987
95% CI	[1.062, 1.085]	[1.093, 1.122]	[1.069, 1.097]	[1.074, 1.093]	[1.045, 1.070	[1.023, 1.097]	[1.014, 1.058]	[1.056, 1.164]	[0.988, 1.095]
Relative accuracy and precision									
Mean bias (% RE)	−1.58	−1.9	0.93	−3.71	−1.38	−2.6	−3.26	4.41	1.08
Repeatability (% CV)	4.53	3.96	4.48	4.71	3.97	6.73	9.49	21.13	16.09
Intermediate precision (% CV)	6.52	4.2	7.71	6.8	4.75	8.66	14.68	36.3	19.9
Robustness									
Mean of ratios	1.03	1.02	0.99	0.99	1.01	0.93	0.89	0.80	0.67
SE	0.013	0.016	0.014	0.019	0.013	0.011	0.021	0.020	0.017
95% CI	[1.003, 1.056]	[0.991, 1.055]	[0.966, 1.024]	[0.947, 1.024]	[0.988, 1.040]	[0.908, 0.953]	[0.850, 0.938]	[0.764, 0.845]	[0.640, 0.710]

**TABLE 3:** IgA assay qualification parameters

Parameter	IpaB	IpaC	IpaD	IpaH	VirG	*S. flexneri* 2a LPS	*S. sonnei* LPS	*S. flexneri* 3a LPS	*S. flexneri* 6 LPS
Limits of quantitation									
LLOD (ECL signal)	1.83	2.40	1.97	0.32	2.10	50.76	13.42	38.99	22.07
LLOQ (ng/mL)	0.183	0.240	0.197	0.032	0.210	5.076	1.342	3.899	2.207
Linearity									
Slope	0.99	1.03	0.98	0.99	1.00	1.17	1.12	1.18	1.06
Coefficient of determination (*R*^2^)	0.994	0.995	0.998	0.995	0.997	0.998	0.998	0.998	0.994
95% CI	[0.956, 1.032]	[1.001, 1.067]	[0.961, 1.002]	[0.961, 1.024]	[0.980, 1.024]	[1.132, 1.199]	[1.099, 1.144]	[1.154, 1.202]	[1.016, 1.102]
Relative accuracy and precision									
Mean bias (% RE)	6.61	9.31	17.65	4.42	14.53	14.9	19.2	18.21	19.84
Repeatability (% CV)	4.35	3.99	6.17	4.66	4.69	13.16	7.83	7.23	14.72
Intermediate precision (% CV)	16.1	11.14	12.46	22.42	12.54	22.38	8.62	17.22	19.03
Robustness									
Mean of ratios	0.80	0.83	0.98	0.84	0.97	1.30	0.97	1.01	1.37
SE	0.022	0.015	0.030	0.010	0.024	0.063	0.033	0.041	0.054
95% CI	[0.759, 0.849]	[0.800, 0.863]	[0.923, 1.045]	[0.823, 0.865]	[0.919, 1.017]	[1.173, 1.430]	[0.902, 1.037]	[0.928, 1.094]	[1.262, 1.484]

#### Assay specificity

**Fig 2:**
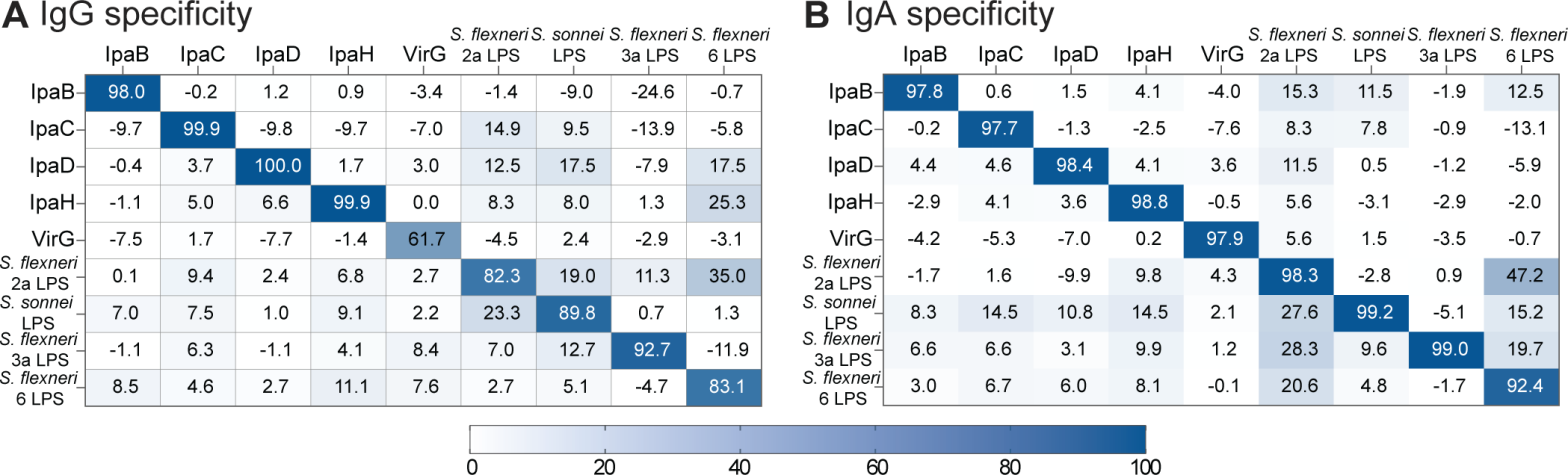
Assay specificity. Heatmaps show percent inhibition of antigen-specific IgG (**A**) or IgA (**B**) binding recorded as ECL signal (columns) of in-house IgG standard pre-incubated with the indicated antigens (rows).

The capacity of the assay to detect antibodies specific to the target antigen was evaluated through binding inhibition experiments. Pre-incubation of the in-house IgG standard (pooled serum) with target antigens resulted in >80% inhibition of the calculated IgG concentration for all antigens except for VirG (61.7%). Although lower than expected, this was the highest inhibition of VirG signal achieved with any of the antigens tested, thus confirming specificity (Fig. 2A). Non-specific antibody binding of the pooled serum was low for all antigens (<25%), except for *S. flexneri* 6 LPS following pre-incubation with *S. flexneri* 2a LPS (35%). This observation is consistent with a low level of cross-reactivity among LPS molecules despite structural differences, which has been observed by others (27, 28).

Assay specificity was greater for the IgA assay, with >92% inhibition of the calculated IgA concentration for all antigens (Fig. 2B). Similar to what was observed for IgG, non-specific antibody binding was <25% for most antigens except for *S. flexneri* 6 LPS when pooled serum was pre-incubated with *S. flexneri* 2a LPS (47.2%), and to *S. flexneri* 2a LPS when pre-incubated with *S. sonnei* LPS (27.6%) and *S. flexneri* 3a LPS (28.3%). These results are in line with the low level of LPS cross-reactivity mentioned above.

#### Assay linearity

Slopes corresponding to linear regressions of log-transformed expected vs calculated IgG and IgA concentrations of 7 mock samples spanning the assay range created by diluting calibrated control samples (with known antibody content) with negative serum ranged between 0.98 and 1.18; coefficient of determinations ranged between 0.987 and 1.000, with 95% CI between 0.956 and 1.202 for both IgG and IgA against all specificities (Tables 2 and 3; Fig. S1). Antibody recovery from serially diluted samples was within the 80%–120% (acceptable) range. Regression analysis of expected vs calculated concentrations for serial dilutions of the in-house standard also exhibited excellent dilutional linearity (*R*^2^ ≥0.987), with consistent linear dose responses (ECL vs dilution factor, Fig. S2).

These results affirm that the capacity of the assay to determine IgG and IgA concentrations is directly proportional to the antibody content of the test sample.

#### Parallelism

Parallelism was examined by comparing the IgG and IgA regression curves for 2–3 experimental samples with the curves of the respective in-house standards. The slopes were not statistically significantly different from each other (Fig. S2A and B), supporting testing of a single dilution and interpolation of antibody concentrations using the standard curve.

#### Accuracy and precision

The relative accuracy and precision were determined by computing the mean bias (% relative error [RE]), repeatability (intra-assay precision), and intermediate (inter-assay) precision of IgG and IgA concentrations assigned to samples representing various antibody content (high, med, low, and very low or near LLOQ) (Tables 2 and 3).

For the IgG assay, the RE was <4.5% for all antigens tested, and the coefficient of variation (CV) in measures of repeatability and intermediate precision was <25% for all antigens except for *S. flexneri* 3a LPS (36.3%). For the IgA assay, the RE ranged between 4.42% and 19.84% for all antigens tested, and the CV for intermediate precision was <25% for all antigens.

Thus, the assay exhibited acceptable levels of accuracy and intermediate precision (<25%).

#### Robustness

The assay robustness was tested by evaluating differences in serum IgG and IgA titers obtained in real scenarios of common assay variations: (i) testing samples with antibody levels spanning the entire assay range (high, med, low, and very low) in a full plate and using an automatic plate washer vs samples tested in a partial plate with washes performed manually and (ii) testing samples in identical conditions of plates and washers, but using 60- vs 75-minute incubation times. The ratio of antibody titers obtained from the full vs half plate runs ranged from 0.8 to 1.03, except for *S. flexneri* 6 LPS (0.67). The standard error (SE) was <0.021 for all antigens (Table 2). The ratio of antibody titers obtained from varying incubation times ranged from 0.80 to 1.2 for all antigens except *S. flexneri* 2a LPS (1.3) and *S. flexneri* 6 LPS (1.37); the SE was <0.063 for all antigens (Table 3). These results indicate that the assay remained unaffected by these small but deliberate variations and is robust for its recommended use.

### Seroprevalence of *Shigella* antibodies in adults living in the USA, Mali, and Malawi

The multiplex assay described above was first used to discern serum IgG levels in adult populations from contrasting geographical areas: USA, Mali, and Malawi, the last two being *Shigella*-endemic regions. The samples tested were archived from prior studies (Table S1).

**Fig 3:**
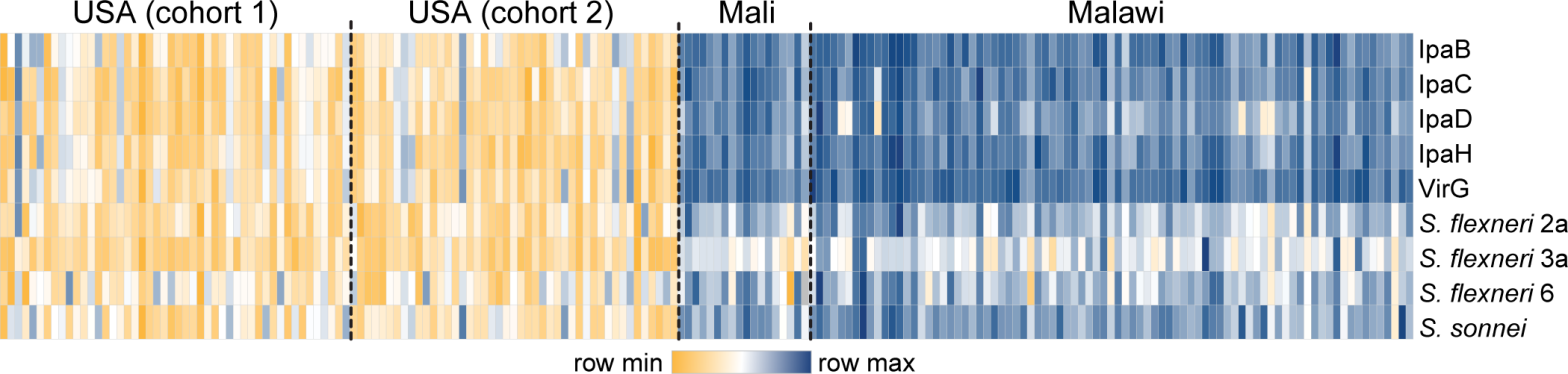
Distribution of *Shigella* antigen-specific IgG levels in adults from different geographical areas. Heatmaps depict IgG antibody profiles in samples from individuals living in the USA (cohort 1, *n* = 28; cohort 2, *n* = 45), Mali (*n* = 18), and Malawi (*n* = 83).

The *Shigella* seroprevalence in the industrialized (USA) vs endemic countries (Mali and Malawi) was strikingly different. Overall, adults from Mali and Malawi exhibited robust levels of IgG against all antigens, much higher in magnitude compared to North American adults (Fig. 3). A small number of US individuals had elevated IgG to protein-specific antibodies. Among LPS specificities, it was notable that a larger proportion of US individuals had high antibody levels against *S. flexneri* 6 and *S. sonnei* LPS, as compared to *S. flexneri* 2a or *S. flexneri* 3a LPS.

### Seroprevalence of *Shigella* antibodies in children living in Mali and Ethiopia

**Fig 4:**
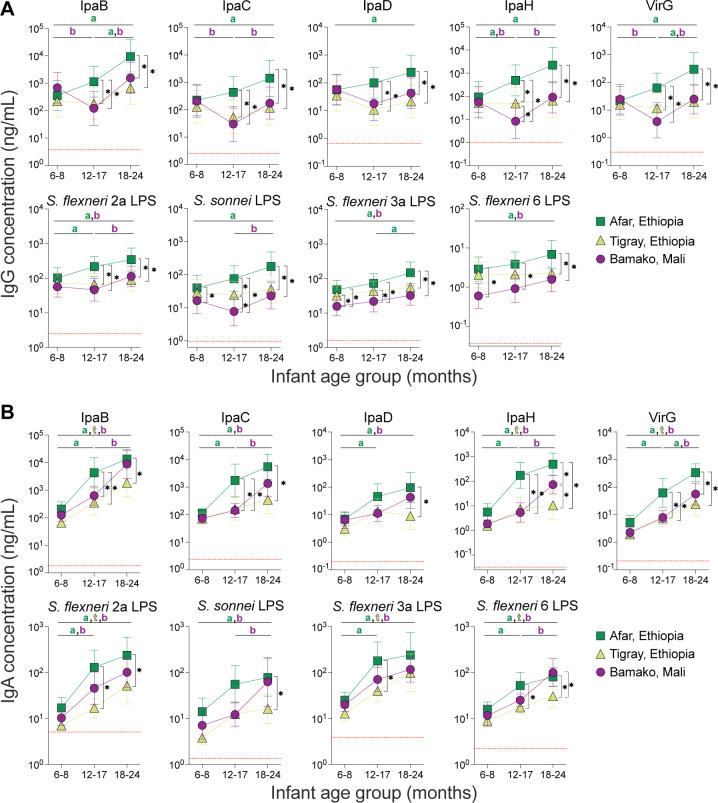
Age-stratified distribution of *Shigella* antigen-specific IgG and IgA titers in children <2 years of age living in endemic areas. Mean IgG (**A**) and IgA (**B**) antibody titers in infants and toddlers representing three age groups (6–8, 12–17, and 18–24 months) living in three regions: Afar (square) and Tigray (triangle), Ethiopia, and Bamako (circle), Mali. Asterisks indicate statistically significant (adjusted *P* value < 0.05) differences in mean titers comparing geographical sites for each age group. Letters indicate statistically significant (adjusted *P* value < 0.05) differences in mean titers between age groups in each site (a = Afar, b = Bamako, and t = Tigray). Means were compared using two-way ANOVA with Bonferroni correction for multiple comparisons. The red dotted line shows the LLOQ for each antigen-specific antibody.

We then examined the serum IgG and IgA specific for *Shigella* antigens in sera from children aged 6 months to 2 years living in three *Shigella*-endemic regions: Bamako in Mali, and Tigray and Afar in Ethiopia (Table 4). Children were grouped into three age categories: 6–8 months, 12–17 months, and 18–24 months, and a cross-sectional analysis of the antibody repertoire was conducted, comparing age groups and geographic sites for each antibody specificity. Mean IgG antibody concentrations for each *Shigella* antigen are shown in Fig. 4A.

**TABLE 4:** Sample numbers and age distribution

Region	Year samples collected	Age range	Mean age	Sex (M/F)	*n* (IgG)	*n* (IgA)
Afar, Ethiopia	2013	6–8 months	7.15	24/21^*a*^	46	22
		12–17 months	13.78	26/19	45	20
		18–24 months	19.37	17/18	35	18
Tigray, Ethiopia	2013	6–8 months	6.79	13/30	43	21
		12–17 months	13.74	16/26	42	19
		18–24 months	19.42	21/17	38	20
Bamako, Mali	2014	6–8 months	6.98	15/29	44	21
		12–17 months	13.89	17/19	36	16
		18–24 months	20.09	21/23	44	20

^*a*^ Sex information not available for 1 individual.

Seroprevalence patterns varied depending on both the geographical site and the children’s age group. Overall, most children had antibodies that recognized all antigens. When comparing the IgG antigenic repertoire in each age group among the different sites, a similar trend was observed for all specificities (Fig. 4A). First, there were no significant differences in antibody levels among the youngest (6–8-months) age group across the three sites, except for IgG levels against *S. sonnei* LPS, *S. flexneri* 3a LPS, and *S. flexneri* 6 LPS, which were higher in Afar or Tigray compared to Bamako. Second, antibody levels in toddlers 12–17 and 18–24 months old living in Afar surpassed those of children from the same age groups living in either Tigray or Bamako. Notably, *S. sonnei* LPS and IpaH IgG levels were significantly different between all sites in the 12- to 17-month-old children, being highest in Afar and lowest in Bamako.

The cross-sectional analysis of antibody levels among age groups once again revealed differences within each of the three sites (Fig. 4A). In Afar, serum IgG titers against all the antigens increased from the youngest to the oldest group, with the antibody levels in the 18- to 24-month group being significantly higher than in the 6- to 8-month group for all specificities. IgG levels in the 12- to 17-month group were higher than the youngest group for IpaH and *S. flexneri* 2a LPS, and between the 12- to 17-month and 18- to 24-month age groups for VirG and *S. flexneri* 3a LPS.

Notably, protein-specific antibody levels in children living in Bamako displayed a V-shaped curve: IpaB, IpaC, IpaH, and VirG IgG titers were significantly lower in the 12- to 17-month-old age group as compared to the 6- to 8-month-old infants, followed by a rise in IgG titers in the 18- to 24-month group. A similar trend, though less clear, was observed for *S. flexneri* 2a, *S. flexneri* 3a, and *S. flexneri* 6 LPS IgG titers, for which levels in the 18- to 24-month group were significantly higher compared to the youngest age group. For *S. flexneri* 2a and *S. sonnei* LPS IgG, antibody levels were significantly higher in children 12- to 17-month-old compared to 6- to 8-month-old infants. In Tigray, any differences in antibody levels among the three age groups did not reach significance (Fig. 4A).

The serum IgA repertoire was likewise impacted by age group and location but displayed different kinetics compared to that of serum IgG (Fig. 4B). When comparing IgA levels among the three age groups, we found that infants in the 6- to 8-month group had the lowest IgA levels for all antigens regardless of geographical site. IgA titers exhibited an upward trend for all antigens in both toddler groups. IgA levels were significantly higher at 12–17 months compared to the 6- to 8-month-old group for *S. flexneri* 2a LPS in Bamako, and for all specificities except *S. sonnei* LPS in Afar. Comparing the two toddler groups, 18- to 24-month-old children in Bamako had significantly higher IgA levels than those in the 12- to 17-month-old group for all specificities except IpaD, *S. flexneri* 2a LPS, and *S. flexneri* 3a LPS. The 18- to 24-month age group had higher IgA levels compared to the youngest age group, reaching significance for IpaB, IpaH, VirG, *S. flexneri* 2a LPS, *S. flexneri* 3a LPS, and *S. flexneri* 6 LPS in all sites, and for IpaC, IpaD, and *S. sonnei* LPS in Afar and Bamako.

Similar to what was observed for IgG, when comparing between sites, protein-specific IgA titers were generally higher in older children from Afar compared to those living in Tigray or Bamako (Fig. 4B). Antibodies against IpaB, IpaC, IpaH, and VirG in 12- to 17-month-old toddlers from Afar were above those from the same age group living in Bamako and Tigray. LPS IgA titers were found to be higher for *S. flexneri* 2a, *S. flexneri* 3a, and *S. flexneri* 6 in the 12- to 17-month age group in Afar compared to those in Tigray. Lastly, IgA titers against all specificities, except *S. flexneri* 3a LPS, in 18- to 24-month-old toddlers in Afar surpassed those of the same age group living in Tigray.

The *Shigella* serological profile contrasted with that of tetanus antitoxin (TT). The kinetics of TT-serum IgG were similar in all three sites, featuring high levels in the 6- to 8-month-old children and a declining trend in the older age groups (Fig. S3). This pattern is consistent with immunization with tetanus-containing vaccines at 6, 10, and 14 weeks of age, as recommended by the Expanded Programme on Immunization (EPI). By contrast, *Shigella* antibodies increased during the first and second years of life, confirming their specificity as secondary to exposure.

**Fig 5:**
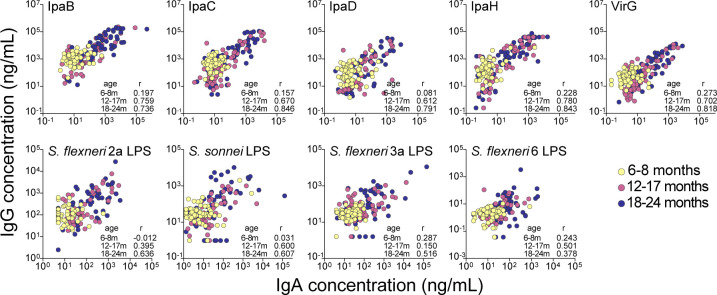
Correlation between *Shigella* antigen-specific serum IgG and IgA antibodies in children. Associations between serum IgG and IgA concentrations against *Shigella* antigens. Data points represent individual antibody concentrations from children from all age groups and geographical regions. Spearman’s *r* values from associations between IgG and IgA concentrations in children stratified by age group are shown within each graph.

Correlation analyses revealed a strong association (Spearman’s *r* > 0.612) between protein-specific IgG and IgA in the 12- to 17- and 18- to 24-month age groups (Fig. 5) but weak associations (if any) in the 6- to 8-month-old infants. Associations between LPS-specific IgG and IgA were less robust than those between protein-specific antibodies but followed the same age-related pattern, particularly for *S. sonnei* and *S. flexneri* 2a LPS.

### Breadth of *Shigella* antibody responses in children

**Fig 6:**
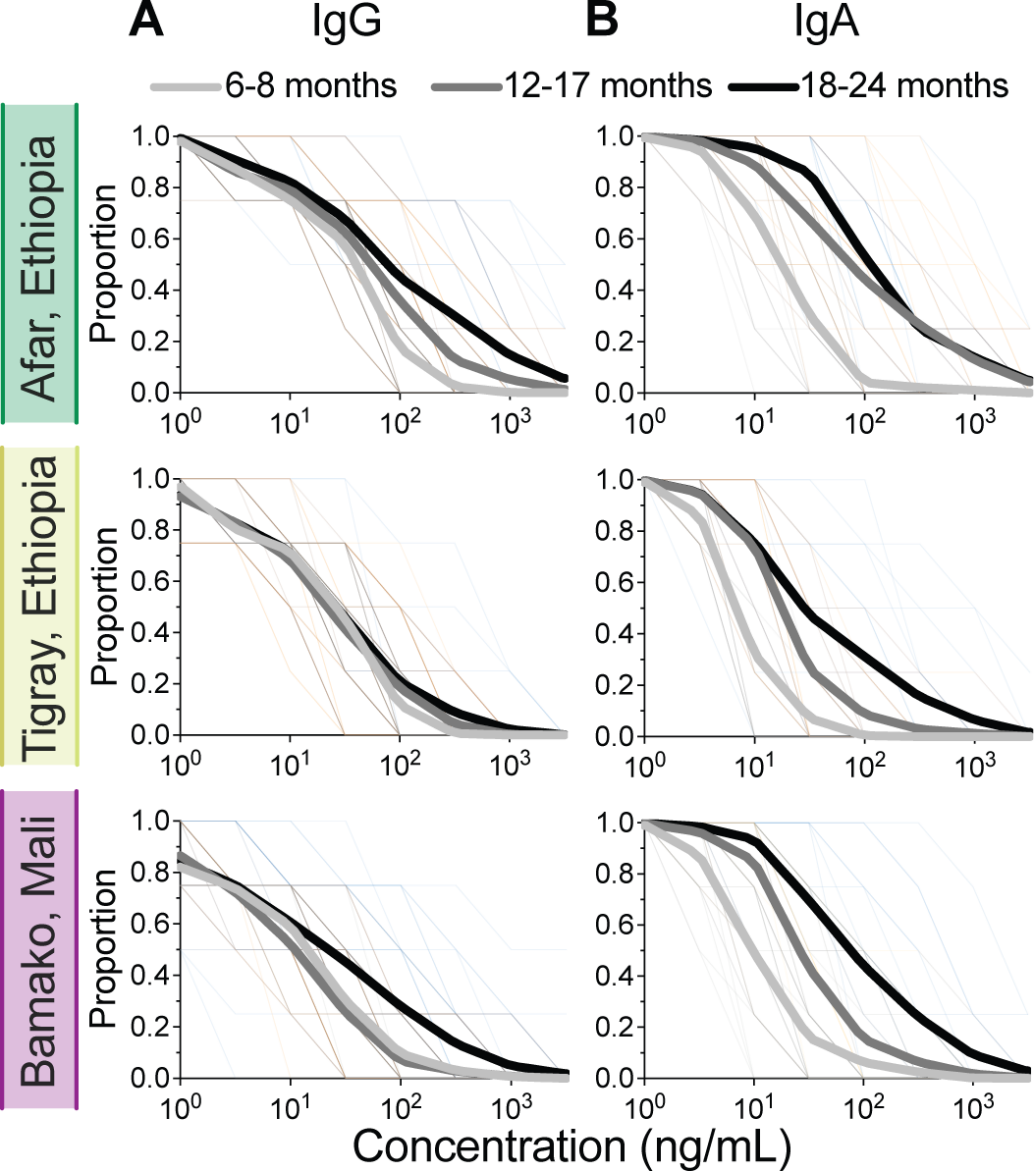
Breadth of *Shigella* LPS antibody repertoire in children. Breadth-potency curves represent the proportion of children in each site with concentrations exceeding a given level (as indicated on the *x*-axis) of IgG (**A**) or IgA (**B**) for any of the 4 *Shigella* LPS serotypes represented on the MSD ELISA (*S. flexneri* 2a, *S. flexneri* 3a, *S. flexneri* 6, and *S. sonnei*). Thick lines represent means for children in each age group, and thin lines represent individual children.

We next examined the breadth of *Shigella* serotype-specific exposure and immunity based on the LPS serological profiles. To do this, we plotted the proportion of children in each age group with LPS antibody levels higher than the indicated concentration (*x*-axis) to generate breadth-potency curves for both IgG and IgA (Fig. 6). Breadth curves from 18- to 24-month-old children were shifted to the right, indicating that this age group had the most expansive repertoire (breadth) of LPS IgG and IgA antibodies across all sites. Site-specific differences were discernible based on antibody class. In Afar, LPS IgG breadth potency increased with the age group but remained the same for all age groups in Tigray. In Bamako, LPS IgG breadth potency was similar in the two younger age groups but increased in the 18- to 24-month-old children (Fig. 6A). By contrast, the breadth of LPS IgA was similar across all three sites, increasing with age group, in Afar (Fig. 6B).

### Associations between *Shigella* antigen-specific antibodies

We also examined associations between antibodies of different specificities to discern links between systemic antibody profiles and immunological priming.

Protein-specific IgG titers were strongly correlated regardless of age group or geographical distribution (Spearman’s *r* ≥ 0.6), with the exception of IpaD in some instances (Fig. S4). Associations between LPS IgG antibodies varied markedly based on age group and site. In the 6- to 8-month-old age group, associations between LPS IgG and protein-specific IgG were generally weak in all sites. In Afar and Tigray, these associations improved in the older age groups except in the 18- to 24-month-old children in Tigray, where associations between *S. sonnei* LPS IgG to any other antigen were low (Spearman’s *r* < 0.4). By contrast, in Bamako, LPS IgG associations with any other antigen remained <0.5 in all age groups; the exception was between *S. flexneri* 2a LPS and the protein antigens IpaB, IpaC, IpaH, and VirG, which were stronger in all age groups (Fig. S4).

Associations between antigen-specific IgA titers did not display any readily apparent patterns between antibody specificities or geographical sites (Fig. S5). Strong associations (*r* > 0.6) were observed between protein-specific IgA levels in 12- to 17-month-old children in Afar and Tigray, and in 18- to 24-month-old children in Bamako. Associations between most other antigen specificities were generally lower, particularly in the youngest age group in Tigray and Bamako.

### Age- and geography-dependent differences in humoral immunity against *Shigella*

**Fig 7:**
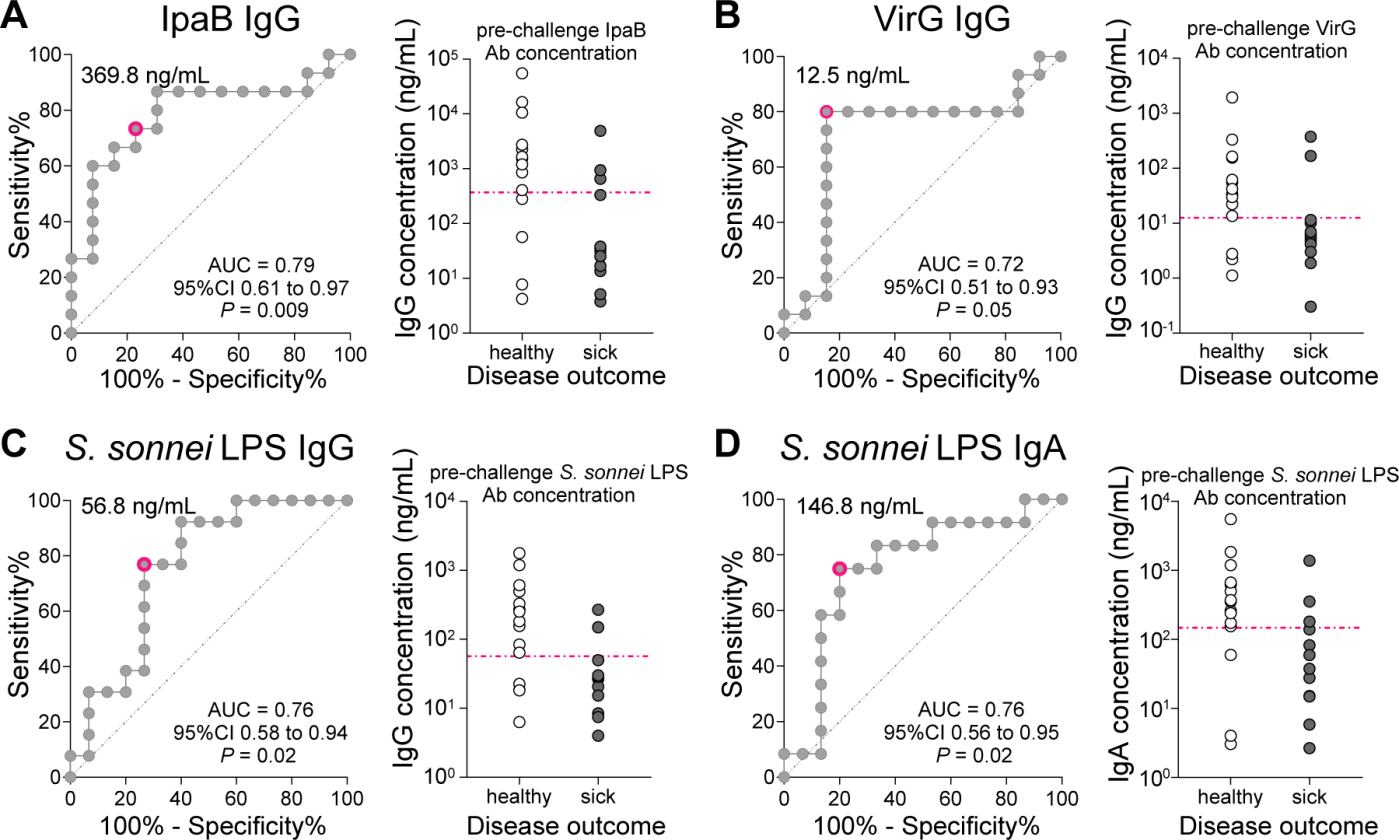
*Shigella* antibody thresholds of protection. ROC curves showing Sensitivity vs 100%—Specificity% calculated using pre-challenge IpaB IgG (**A**), VirG IgG (**B**), *S. sonnei* LPS IgG (**C**), and *S. sonnei* LPS IgA (**D**) antibody concentrations in serum from individuals who remained healthy or had mild disease (healthy) and those who had moderate or severe disease (sick) following the first challenge. AUC, 95% confidence interval (CI), and *P* values are shown in the graph on the right in each panel. Individual antibody titers in healthy and sick volunteers are shown on the graph on the left of each panel; the pink dotted line indicates the optimal threshold value obtained from the corresponding AUC curve.

To interpret the serological profiles observed in the children from different age groups and sites in terms of protection status, we compared their IgG and IgA titers with threshold levels associated with the clinical protection of adults who participated in CHIM studies (29, 30). The CHIM samples were tested using the *Shigella* MSD platform, and threshold levels were computed by comparing pre-challenge titers from subjects who remained healthy (healthy or mild disease) to those who became sick (moderate or severe disease) using receiver operating characteristics (ROC) curves. Optimal protective cut-off values were determined for antibodies with an area under the curve (AUC) significantly greater than 0.5: IpaB IgG (369.8 ng/mL), VirG IgG (12.5 ng/mL), *S. sonnei* LPS IgG (56.8 ng/mL), and *S. sonnei* LPS IgA (146.8 ng/mL) (Fig. 7).

**Fig 8:**
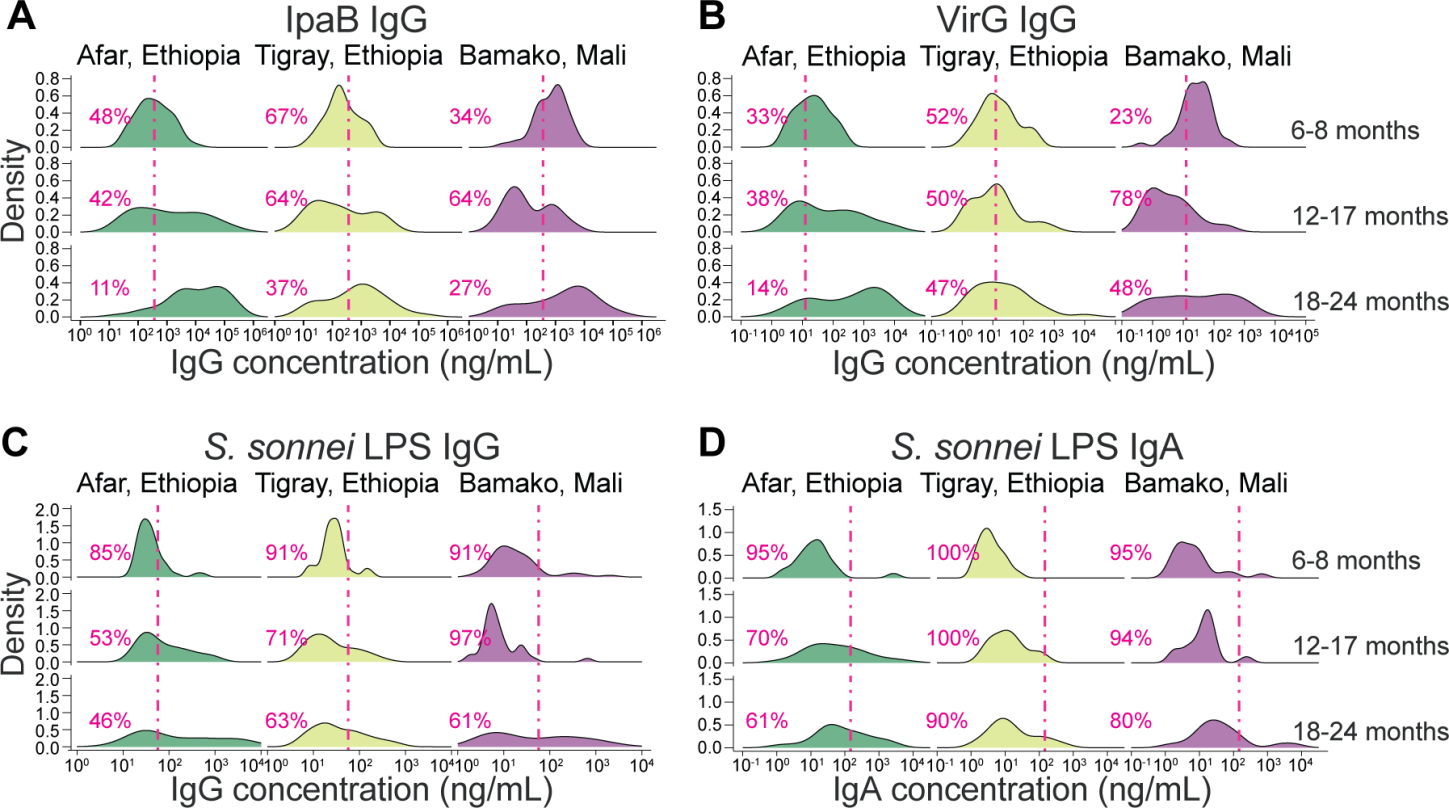
Cross-sectional distribution of serum antibody levels in children. Density plots depict IpaB IgG (**A**), VirG IgG (**B**), *S. sonnei* LPS IgG (**C**), and *S. sonnei* LPS IgA (**D**) distributions among the three age groups in the three sites. The pink dotted line marks the optimal protective threshold based on the ROC analysis curves. The percentage of children with antibody levels lower than the threshold is indicated in each graph.

An analysis of the distribution of IpaB and VirG IgG levels revealed that the proportions of children with levels above the protective cutoff were primarily age dependent and, overall, similar in all three sites (Fig. 8A and B). Most children (>50%) in the 6- to 8-month-old age group in Afar and Bamako had antibody levels greater than the IpaB and VirG IgG thresholds, likely representing residual maternally acquired antibodies (11). The situation was different in Tigray, where 67% and 52% of 6- to 8-month-old infants had IpaB and VirG IgG titers, respectively, below the calculated protective cutoff.

A shift was observed in the 12- to 17-month-old toddlers in Bamako, the majority of whom had IpaB and VirG IgG levels below the protective threshold (64% and 78%, respectively). The majority of 12- to 17-month-old toddlers from Tigray also had IpaB and VirG IgG titers below the protective thresholds. A subsequent and more pronounced shift occurred in 18- to 24-month-olds, when, across all sites, more than 50% of children had acquired IpaB and VirG IgG levels above the protective threshold. Notably, in Afar, almost all children (89% for IpaB IgG and 86% for VirG IgG) had antibody levels above the threshold.

The calculated *S. sonnei* LPS IgG or IgA threshold values were more stringent (Fig. 8C and D); most of the children had levels below the protective threshold, except for the 18- to 24-month-old group in Tigray for which 54% were above the *S. sonnei* LPS IgG cutoff value (Fig. 8C). Nevertheless, the shift described above for protein antibodies in the older toddlers was also apparent for LPS antibodies: the proportion of children with protective titers increased across the age groups and was largest in the 18- to 24-month-old toddlers.

## DISCUSSION

The accurate measurement of biological markers is essential for informed decision-making in clinical medicine. Population-based analyses of antibodies as markers of microbial exposure (known as seroprevalence surveys or serosurveys), in particular, are useful as they provide insights into the circulation of pathogens, immune status of the population, and provide quantitative evidence on disease susceptibility that can guide public health response strategies. In their simplest form, serosurveys are conducted through traditional antibody binding assays. Methods that allow for accurate, sensitive, and quantitative high-throughput analysis of clinical samples with ease are desirable.

Here, we report the development and qualification of a meso scale discovery (MSD) multi-array electrochemiluminescence assay that enables simultaneous quantification of *Shigella* antibodies of multiple specificities in a high-throughput manner. This assay exhibited adequate specificity and sensitivity, a wide dynamic range, and excellent linearity, accuracy, and precision. It requires less than 5 µL of sample, adding the benefit of sample sparing. MSD-based assays have been developed for the analysis of antibodies to various bacterial and viral antigens in vaccine studies (31–33); the platform is applicable to various sample types and widely used for vaccine and clinical immunology.

The *Shigella* field has lagged in the implementation of rigorous, quantitative, high-throughput methods for antibody determination. A multiplex Luminex-based immunoassay developed by Kaminski et al. (34) included 6 *Shigella* protein and LPS antigens coated onto labeled beads. A strong correlation with ELISA data was reported. However, the assay was not analytically characterized, nor was it applied to subsequent studies. An extension of this multiplex bead-based assay is being implemented to explore humoral immunity to *Shigella* in children with PCR-detected acute and convalescent infection using alternative sampling methods (e.g., dry blood spots eluates), although with a limited diversity of antigenic probes (35). Our group (22) and others (36) have used Luminex-based platforms to interrogate multiple features of *Shigella* antibodies, including function. While useful for high-dimensional comparison of antibody binding and functional features in defined groups, these methods lack sensitivity and precision for quantitative analysis of unknown and unrelated samples. The antibody titers are arbitrary (reported in median fluorescent intensity), usually generated from a single dilution, and not comparable between studies.

Improving upon these platforms, in addition to LPS from the most prevalent serotypes and most well-known type III secretion proteins (IpaB, IpaC, and IpaD), our *Shigella* MSD assay included IpaH and VirG, the latter being a protective antigen in animals and putative correlate of protection in humans (21, 37). It was also fully qualified for quantification of both serum IgG and IgA. The MSD remains the preferable platform for strict antibody quantification due to its higher sensitivity, wider dynamic range, and greater accuracy and precision as compared to other multiplex platforms.

Our analysis of *Shigella* seroprevalence in individuals living in endemic areas (Mali and Malawi) as compared to those living in the US revealed heightened antibody levels in the former, consistent with environmental exposure. Importantly, quantitative endpoints were generated over a wide dynamic range (representing vast differences in antibody content in the two populations) with a single dilution scheme. Thus, the MSD assay proved to be adequate to distinguish serological profiles of individuals living in different settings and with diverse histories of pathogen encounter.

There is no vaccine approved to prevent *Shigella* dysentery. A successful candidate would need to be effective in children under 3 years of age, for whom the risk of infection is highest (7, 24). The World Health Organization (WHO) preferred product characteristics for *Shigella* vaccines call for a target age starting at 6 months (38). There is limited understanding of the evolution of immunity during the critical first 6–24 months of life and the elements of immunity that would prevent infection in early life. Our study uniquely mapped out the prevalence of *Shigella* antibodies in children aged 6 months to 2 years from Bamako in Mali and Tigray and Afar in Ethiopia (39). All children had circulating antibodies against *Shigella* proteins and LPS antigens, yet their magnitude and dynamics varied according to age and geographical location.

The presence of IgG in the 6- to 8-month-old children reflects the robust humoral maternal immunity, and the similarity in IgG levels among the three sites (particularly against the protein antigens) indicates the common processes of placental transfer and antibody decline over time, independent of geographical site. Thompson et al. examined the decay of *S. sonnei* IgG antibodies in infants born to Vietnamese mothers and reported an estimated half-life of 43 days with a steady decline over the first year of life (10). This passively acquired maternal immunity contributes to the early shielding of disease in young infants.

Geographical region had a major impact on the children’s serological profile with increasing age. In Bamako, IgG levels sharply declined in 12- to 17-month-olds and markedly increased in 18- to 24-month-olds, consistent with waning of maternal protection and accrual of immunity with increasing diarrheal episodes. Similar waning of *Shigella* antibodies during the first year of life had been reported in studies conducted in Vietnam (10), Zambia (12), and Kenya (16). Notably, our seroprevalence data revealed differences between the two sites within Ethiopia. Children from Afar had higher antibody levels which developed sooner compared to those from Tigray, indicative of earlier exposure to *Shigella* (by 12–17 months). In fact, in Afar, antibody levels increased linearly (from the youngest to the oldest age group), while in Tigray, *Shigella*-specific IgG developed more gradually showing no differences with age. These variations are attributable to lifestyle differences: compared to the more urban and agrarian Tigray region, populations living in the Afar region are predominantly nomadic and more vulnerable to food- and water-borne pathogens like *Shigella*. Children from Tigray are more likely to have access to basic sanitation and healthcare facilities (39, 40). Furthermore, children in Afar had the highest LPS IgG antibody breadth potency, suggesting a wider exposure to *Shigella* serotypes compared to children living in Tigray or Bamako. In line with our seroprevalence data, a recent study reported *S. sonnei* IgG seroincidence rates being highest at 12–18 months in a longitudinal study of children 12–60 months old in Vietnam (41).

Unlike previous serosurveys, which circumscribed analysis of antibodies to *Shigella* LPS (10, 12, 16), our study profiled both serum IgG and IgA antibodies to *Shigella* protein antigens. The steady increase of serum IgA—higher in the 12- to 17-month-olds compared to the 6- to 8-month-olds, and even higher in the 18- to 24-month group—stands in sharp contrast to the kinetics of serum IgG and points to IgA in circulation as a unique biomarker of *Shigella* exposure during the first years of life. The increase in IgA in 12- to 24-month-old children is consistent with the higher incidence of *Shigella* in this age group reported in previous multi-site studies including the Global Enteric Multicenter Study (GEMS) (7) and Vaccines Impact on Diarrhea in Africa (VIDA) (24). A similar increase in *S. flexneri* 2a and *S. sonnei* LPS IgA levels was observed in Zambian infants during their first year of life (12).

Strong associations were found between protein-specific IgG and IgA in children aged 12–24 months. Associations between LPS-specific IgG and IgA were more modest but showed improvement in the 18- to 24-month group. This delay in the acquisition of LPS-specific antibodies, compared to protein-specific antibodies, suggests mechanistic differences in immunological priming, with activation of different types of B cells based on antigen type. The correlative analysis reaffirmed the more robust immunogenic capacity of *Shigella* proteins over LPS along with the coordinated production of both antibody types as a result of mucosal exposure. Protein- and LPS-specific antibodies play different and complementary roles in protection depending on the environment and biological scenario. For example, in studies in North American individuals, both neutrophil phagocytosis mediated by IpaB (22) and bactericidal activity (21, 42), which is facilitated by LPS antibodies (11), were associated with clinical protection. On the other hand, *S. flexneri* 2a OPS IgA-dependent binding to FcαR and neutrophil phagocytosis were associated with resistance to shigellosis within 90 days in a high *Shigella* burden (military) area in Peru (36).

Gaps in knowledge remain regarding the attributes of naturally acquired immunity that prevent *Shigella* infection, including (i) precise antigenic targets (protein specific vs LPS specific), (ii) type of antibodies induced, (iii) life-long evolution from infancy through adulthood, (iv) longevity and boosting stimuli, and (v) sites and functional mechanisms of action involved.

In the absence of precise indicators of protective immunity, we used multiplex serological data from a *Shigella* CHIM study to establish antibody thresholds associated with clinical protection in human adults. We hypothesized that antibodies against IpaB and VirG contribute to preventing *Shigella* infection in humans and determined the proportion of children achieving IpaB and VirG serum IgG protective levels. This analysis revealed that the most vulnerable were children in the 6- to 8-month or those in the 12- to 17-month age group, depending on the site. The multiplex CHIM data allowed us also to discern *S. sonnei* LPS IgG and IgA levels as putative correlates of protection against *S. flexneri* 2a challenge. *S. sonnei* LPS IgA antibodies were previously identified as protective against homologous *S. sonnei* challenge (42). Intriguingly, *S. sonnei* challenge has also been shown to provide protection against heterologous *S. flexneri* 2a challenge in naïve American volunteers (43). *S. sonnei* LPS thresholds were more stringent, and children 18–24 months of age had the highest proportion of children above the IgG and IgA protective cutoffs. Collectively, these findings emphasize the vulnerability to infection during the first 2 years of life until sufficient adaptive immunity develops.

Our results are limited in that they portray a serological landscape of exposure to *Shigella* and not necessarily of protective immunity. Prospective studies that combine active surveillance of *Shigella* infection with in-depth immunological interrogations are needed to identify correlates of protection specific for the under-3 age group; identification of such markers is essential to interpret naturally acquired immunity and to predict vaccine efficacy. Immunological correlates against *Shigella* have remained elusive, impeding vaccine development and increasing reliance on CHIM studies to identify effective candidates.

One challenge that prevents attaining the full potential of serological assays is the lack of calibrated standards that would allow the comparison of serological readouts across studies and laboratories. An internationally calibrated standard with adequate levels of antibodies of multiple specificities (as opposed to uncharacterized convenience samples) will help standardize and facilitate sample testing and expedite vaccine evaluation efforts.

In summary, we have developed a reliable, simple, and sample-sparing multiplex assay for the quantification of *Shigella* antibodies suitable for seroepidemiological studies, evaluation of immune responses in vaccine and CHIM studies, and for evaluation of vaccine introduction and effectiveness. This platform could be useful to discriminate between susceptible, infected, and protected individuals, especially in studies involving young children, the target group for *Shigella* vaccines. The analysis of serological profiles in children 6 months to 2 years of age revealed epidemiological trends and identified children 12- to 17-month-old as the most vulnerable to infection. Increased levels of *Shigella* protein-specific antibodies in older and less vulnerable children affirm their potential as vaccine candidates.

## MATERIALS AND METHODS

### Clinical samples

For this study, we included serum samples archived in repository freezers at the Center for Vaccine Development and Global Health (CVD) at the University of Maryland School of Medicine. Serum samples from North American individuals included (i) pre-challenge samples from a controlled human infection study (CHIM) of wild-type *S. flexneri* 2a strain 2457T as described previously (29) and (ii) an immune status surveillance study performed at the CVD in 2015 (Table S1). Serum samples from Malian adults were a randomly selected subset of mothers who participated in a maternal influenza study (44); those from Malawi were from a malaria surveillance cohort in Chikwawa, Malawi (11). Sera from mothers in the Malawi cohort were used to generate in-house standards and controls (Table S1).

To assess *Shigella* seroprevalence in children, we used archived samples from children aged 6–24 months. Samples were obtained from Ethiopian children enrolled in immunization coverage surveys between February and April 2013 in Afar (Assaieta woreda) and Tigray (Hintalo Wajerate woreda) (39, 45). Malian samples were from a field evaluation of a rapid assessment tool to detect protective titers against tetanus antitoxin in infants and toddlers in various sites in Bamako in 2017 (unpublished data). Clinical samples were consented for future use.

### Antigens and standards

*Shigella* antigens IpaB, IpaC, IpaD, and LPS from *S. flexneri* 2a, *S. flexneri* 3a*, S. flexneri* 6, and *S. sonnei* were obtained from Walter Reed Army Institute of Research (WRAIR). The N-terminal domain of VirG was expressed and purified in an *E. coli* expression system (37). IpaH was produced by Vaxcyte Inc. using a cell-free expression system (46). An in-house serum IgG standard was generated by pooling equal volumes of sera from five mothers from Malawi that had high titers by traditional ELISA (11). An IgA standard was generated by pooling equal volumes of breast milk from 80 mothers from the same Malawi cohort. In addition, two high titer serum samples from the same Malawi cohort were pooled to produce the in-house positive control. Human serum minus IgA/IgM/IgG (Sigma-Aldrich, St. Louis, MO) was used as negative control.

### MSD multiplex assay

#### Calibrating the in-house standard

*Shigella*-specific IgG and IgA antibody concentrations of our in-house standards were determined using a method described by Zollinger et al*.* (26). Briefly, Immulon 2HB plates (Thermo Scientific, Waltham, MA) were coated with IpaB, IpaC, IpaD, or IpaH at 0.1 µg/mL in PBS, VirG at 2.5 µg/mL in PBS, and all LPS antigens at 5 µg/mL in carbonate buffer, pH 9.6. Plates were incubated for 3 h at 37°C and blocked at 4°C overnight in PBS containing 10% wt/vol non-fat dry milk (NFDM). The standards were diluted in PBS containing 10% NFDM and 0.05% Tween-20 (PBS-T) and added to the plates. After a 1 h incubation at 37°C, plates were incubated with HRP-labeled goat secondary antibody (Jackson Immuno Research, West Grove, PA) specific for human IgG (Fc) or IgA (α-chain specific) for another 1 h at 37°C. Plates were washed six times with PBS-T following every incubation step. Tetramethylbenzidine (TMB; KPL, Gaithersburg, MD) was added as a substrate for 15 min in the dark with shaking (700 rpm), and the reaction was stopped by adding 1M phosphoric acid (Millipore Sigma, Burlington, MA). Antigen-specific antibody concentrations were extrapolated from standard curves generated by serial dilution on purified human IgG or IgA (MP Biomedicals, LLC, Irvine, CA) of known concentrations tested on the same plate.

#### Optimization of antigen coating conditions

Optimization and antigen coating of plates were performed according to the manufacturer’s protocols (Meso Scale Diagnostics, Rockville, MD). Initial tests were conducted with the MSD optimization package to determine the optimal coating conditions. The package included U-PLEX 96-well plates (with up to 10 spots per well) printed with the nine antigens on spatially distinct spots per well, at different coating concentrations (20, 50, and 100 µg/mL), and using two different coating buffers (PBS + stabilizer and PBS + stabilizer + BSA). Serial threefold dilutions of the in-house positive control were tested on plates for each condition. The ECL signals obtained at the highest, mid, and lowest dilutions were compared to the ECL signal obtained for each antigen with the blank to determine a signal-to-noise ratio. Coating conditions yielding high signal-to-noise ratios (>8,000 for protein antigens and >200 for LPS antigens) with ECL signal values for dilutions of standards within the range of the assay (1,000–1,000,000) were chosen. These were determined to be 50 µg/mL for IpaB, IpaC, IpaD, VirG, IpaH, *S. flexneri* 2a LPS, *S. flexneri* 3a LPS, and *S. sonnei* LPS and 100 µg/mL for *S. flexneri* 6 LPS. Plates were custom printed by MSD, shipped, and stored at 4°C until use; the same batch of plates was used for all assay qualification experiments.

#### MSD assay

Plates were blocked with PBS with 0.05% Tween-20 (PBS-T) containing 10% NFDM (assay diluent 1). In-house standard, control, and test samples diluted in assay diluent 1 were added. After incubation and washing, biotinylated goat anti-human IgG (Jackson ImmunoResearch) diluted in PBS-T with 1% bovine serum albumin (Millipore) (assay diluent 2) was added as secondary antibody, after which the detection antibody (SULFO-TAG Streptavidin) in assay diluent 2 was added. All incubation steps were at room temperature for 1 h with shaking. Plates were washed three times with PBS-T between incubation steps. MSD GOLD Read Buffer A was added, and plates were read using an MSD QuickPlex SQ 120 reader within 20 minutes. Data were analyzed using MSD Discovery Workbench Version 4.0 (MSD, Rockville, MD). ECL signals were automatically adjusted by subtracting the blank signal. Curves and curve fit statistics (*R*^2^ and hillslope) were generated for each of the standards using a 4-parameter logistic fit (4PL) by the MSD Workbench software.

The in-house standard curves for each antigen were generated using 8 threefold serial dilutions, starting at a 1:10,000 for IgG (Fig. 1A) and 1:100 for IgA (Fig. 1B). The in-house standards, in-house positive control (1:30,000 for IgG and 1:6,000 for IgA), and negative control (1:100) (described above) were included in all assays.

Clinical serum samples were initially tested at 1:2,000 dilution for IgG and 1:300 for IgA, and at a higher or lower dilution if the ECL did not fall within the in-house standard detection range. The minimum required dilution (MRD) in our study was set at 1:100. Antibody concentrations of study samples and controls were determined using MSD Discovery Workbench and reported in ng/mL.

### Assay qualification

#### Assay specificity

To confirm assay specificity, the in-house IgG serum standard, diluted at 1:40,000 for the IgG assay and 1:6,000 for the IgA assay, was pre-incubated with 10 μg/mL of each of the nine individual antigens at room temperature for 1 h before testing in the MSD assay. ECL values of the adsorbed sample preparations were compared to those of non-adsorbed samples. Specificity for both homologous and heterologous antigens was expressed as percent inhibition as determined by the equation: 100 − (adsorbed sample ECL/non-adsorbed sample ECL) × 100. The averaged percent inhibition values between two runs are reported in Fig. 2.

#### Limits of quantitation

The lower and upper limits of detection (LLOD and ULOD, respectively) for each antigen were determined from the individual standard curves and are shown in Tables 2 and 3. The lower limit of quantification (LLOQ) was calculated by multiplying the LLOD by the MRD (Tables 2 and 3).

#### Linearity and parallelism

To evaluate assay linearity, mock samples were prepared by diluting serum samples with assigned antibody concentrations through 5–7 dilution factors in negative serum (Fig. S1). Linear regression analysis of log-transformed calculated vs expected antibody concentrations was performed. The slope, coefficient of determination (*R*^2^), and standard deviation of residuals (Sy.x) were determined (Tables 2 and 3). Dilution linearity was determined by performing linear regression analysis on calculated antibody concentrations vs expected antibody concentrations following threefold serial dilutions of the in-house serum standard, starting at 1:10,000 for IgG, through 6–8 dilution factors. The slope, coefficient of determination (*R^2^*), and standard deviation of residuals (Sy.x) were determined. The log-transformed ECL signal was plotted vs log-transformed dilution factor to examine dilutional linearity.

To determine parallelism, slopes calculated from threefold serial dilutions of serum samples obtained from three or four volunteers were compared to that of the in-house standard (Fig. S2). *P* values > 0.05 were considered to indicate that the slopes were parallel and not significantly different from each other.

#### Relative accuracy and precision

Relative accuracy was measured by calculating the mean bias, repeatability, and intermediate precision (Tables 2 and 3). “Mock” samples were prepared by pre-diluting the in-house serum standard with negative serum as follows: high (neat sera); mid (1:2), low (1:8) and very low (1:32); or near LLOQ (1:512). These samples were tested in five independent assay runs performed by two different operators on two different days, which generated *n* = 40 datapoints for each dilution level. Mean bias, expressed as % relative error (%RE), was calculated as follows: %RE = [(calculated concentration/nominal concentration) − 1] × 100 (47). Intra-assay %CV (repeatability) and inter assay %CV (intermediate precision) were also calculated.

#### Robustness

We tested assay robustness by evaluating differences in plate washing methods (Table 2) or antibody incubation times (Table 3). Four samples at high, mid, low, and very low (or near LLOQ) concentrations were run on two different plates in the same way for all assay steps, varying either the washing procedure (automatic plate washer vs manual wash) or incubation times (60 minutes vs 75 minutes). The calculated concentrations of each sample were then compared between the two plates.

### Statistical analysis

Linearity and parallelism were assessed by linear regression analysis. Mean bias (%RE), intra- and inter-assay %CV were calculated based on a one-way analysis of variance (ANOVA), as previously described (47). Heat maps were created using GraphPad Prism version 9 or MORPHEUS (https://software.broadinstitute.org/morpheus). Comparison of antibody levels among infant age groups at the three geographical sites (Fig. 4) was performed by two-way ANOVA with Bonferroni correction for multiple comparisons. Adjusted *P* values < 0.05 were considered statistically significant. Breadth-potency curves were generated by plotting the proportion of individuals with LPS specificity above a given concentration (*x*-axis) and fitted by LOWESS smoothing in GraphPad Prism version 9 (GraphPad, San Diego, CA). ROC analysis was performed using data from pre-challenge antibody concentrations from individuals who remained healthy (healthy or mild disease) vs those who were sick (moderate or severe disease [29, 30]). Optimal antibody concentration thresholds were estimated using the nearest to (0,1) method (48, 49), with sensitivity and specificity >70%. Density plots (Fig. 8) were created using the geom_density function (ggplot2 package) in R version 4.4.0, as previously described (50). Statistical analyses were performed using MSD Discovery Workbench, GraphPad Prism version 9 (GraphPad, San Diego, CA), or Stata/SE Version 18 (StataCorp, College Station, TX).

## SUPPLEMENTAL MATERIAL

Supplemental material mbio.00555-25-s0001.pdf Supplemental figures and table.

ASM does not own the copyrights to Supplemental Material that may be linked to, or accessed through, an article. The authors have granted ASM a non-exclusive, world-wide license to publish the Supplemental Material files. Please contact the corresponding author directly for reuse.
