# Supplementary material for: Shigella humoral immunity during the first 2 years of life in children from endemic areas
Source: mBio. 2025 Apr 16;16(5):e00555-25. doi: 10.1128/mbio.00555-25 (PMC12077217; doi:10.1128/mbio.00555-25)
Supplement: Supplemental material — Supplemental figures and table. [file mbio.00555-25-s0001.pdf]

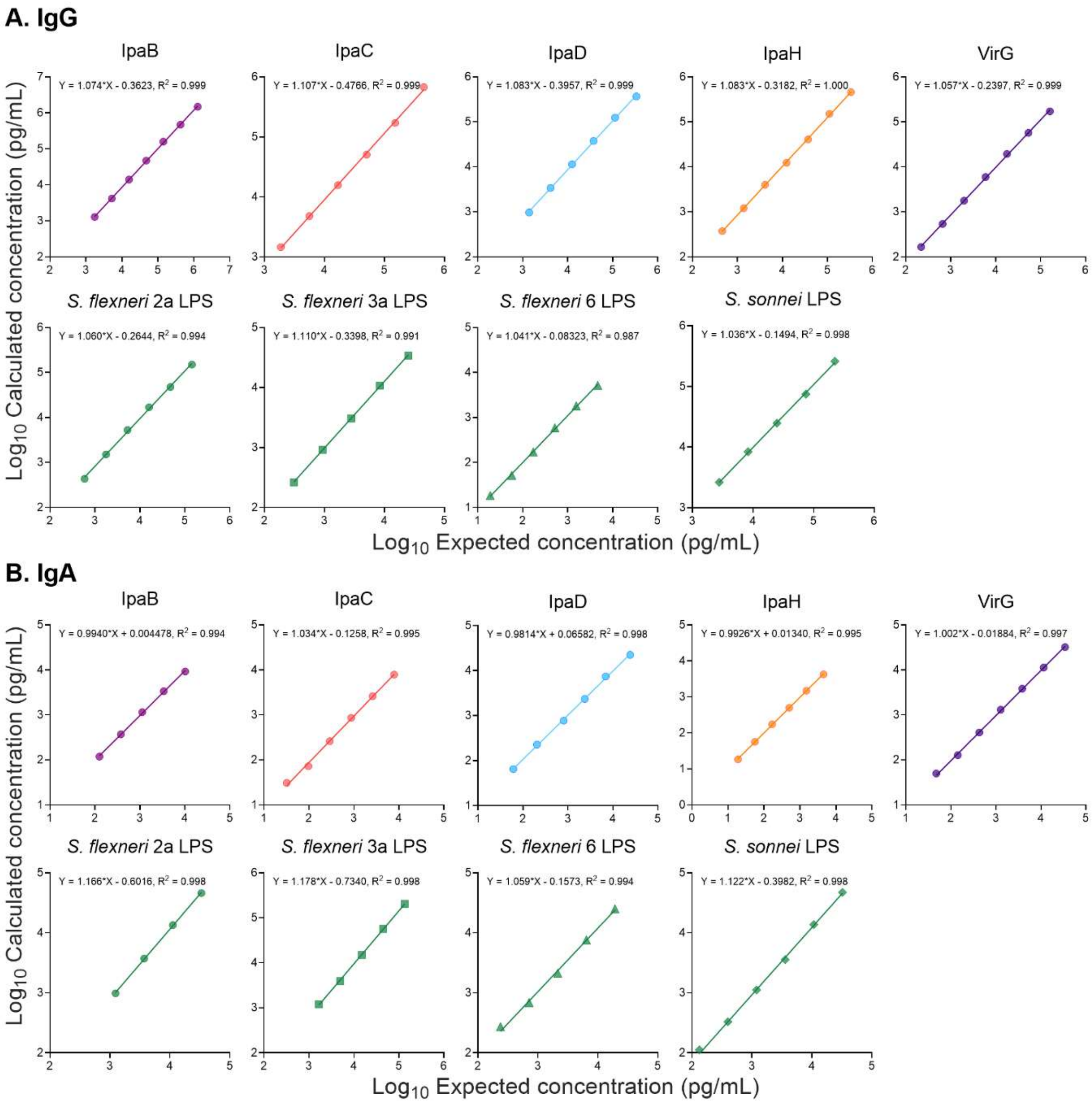

Figure S1. Assay linearity. Representative mock samples generated by dilution of pooled serum controls in negative serum were tested in the MSD IgG (**A**) and IgA (**B**) assay. Log-transformed calculated (y-axis) vs expected (x-axis) antibody concentrations at each dilution were compared. Linear regression curves with the equation and R^2 values for each antigen are shown.

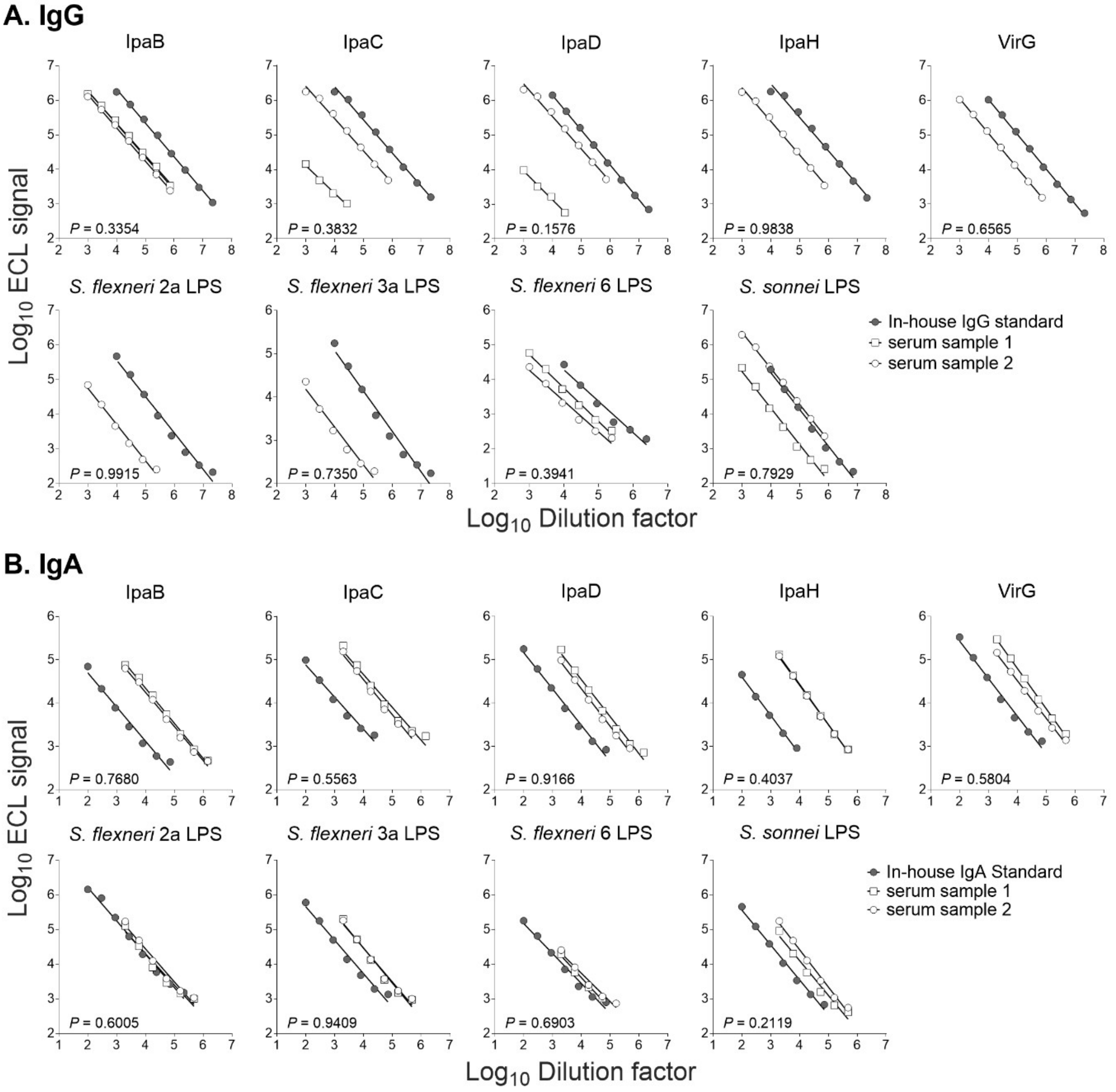

Figure S2. Parallelism. The in-house standards and 2 serum samples from different donors were tested in three-fold serial dilutions in the MSD IgG (**A**) and IgA (**B**) assays. Log-transformed ECL signals were plotted against the dilution factor; linear regression curves are shown for each sample in each graph. Comparisons between the lines showed that the slopes were not significantly different (*P*>0.05).

Table S1. Adult cohort characteristics

	Year samples collected	Age range	Sex (M/F)	n
USA (cohort 1)[1]	1993	18-40 years	26/2	28
USA (cohort 2)	2015	22-62 years	14/31	45
Mali[2]	2011	15-39 years	0/18	18
Malawi[3]	2016	17-43 years	0/18	83

[1]Kotloff et al., 1995. Evaluation of the safety, immunogenicity, and efficacy in healthy adults of four doses of live oral hybrid *Escherichia coli- Shigella flexneri* 2a vaccine strain EcSf2a-2. Vaccine 13:495-502.

[2]Tapia et al., 2016. Maternal immunisation with trivalent inactivated influenza vaccine for prevention of influenza in infants in Mali: a prospective, active-controlled, observer-blind, randomised phase 4 trial. Lancet Infect Dis doi:10.1016/S1473-3099(16)30054-8. doi:10.1016/S1473-3099(16)30054-8

[3]Ndungo et al., 2021. Repertoire of Naturally Acquired Maternal Antibodies Transferred to Infants for Protection Against Shigellosis. Front Immunol 12:725129. doi:10.3389/fimmu.2021.725129

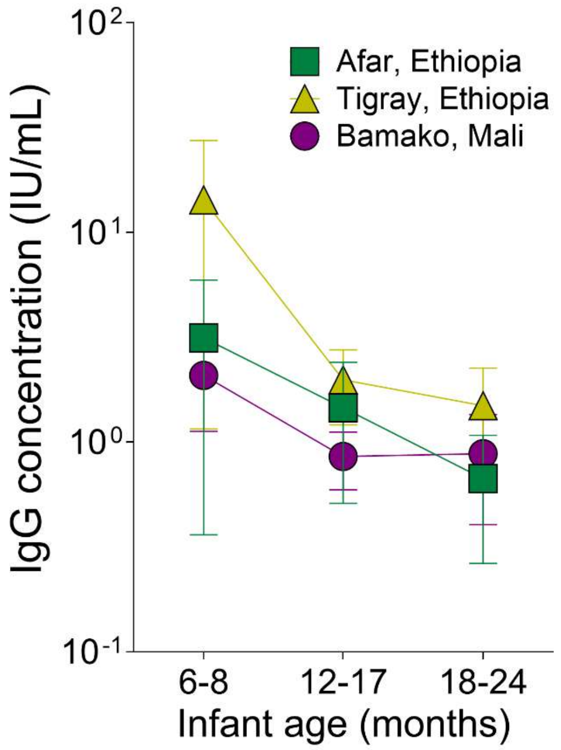

Figure S3. Age-stratified distribution of mean tetanus toxoid IgG in children <2 years of age. Mean IgG antibody levels in infants and toddlers representing three age groups (6-8, 12-17, and 18-24 months) living in three regions: Afar (square) and Tigray (triangle), Ethiopia and Bamako (circle), Mali.

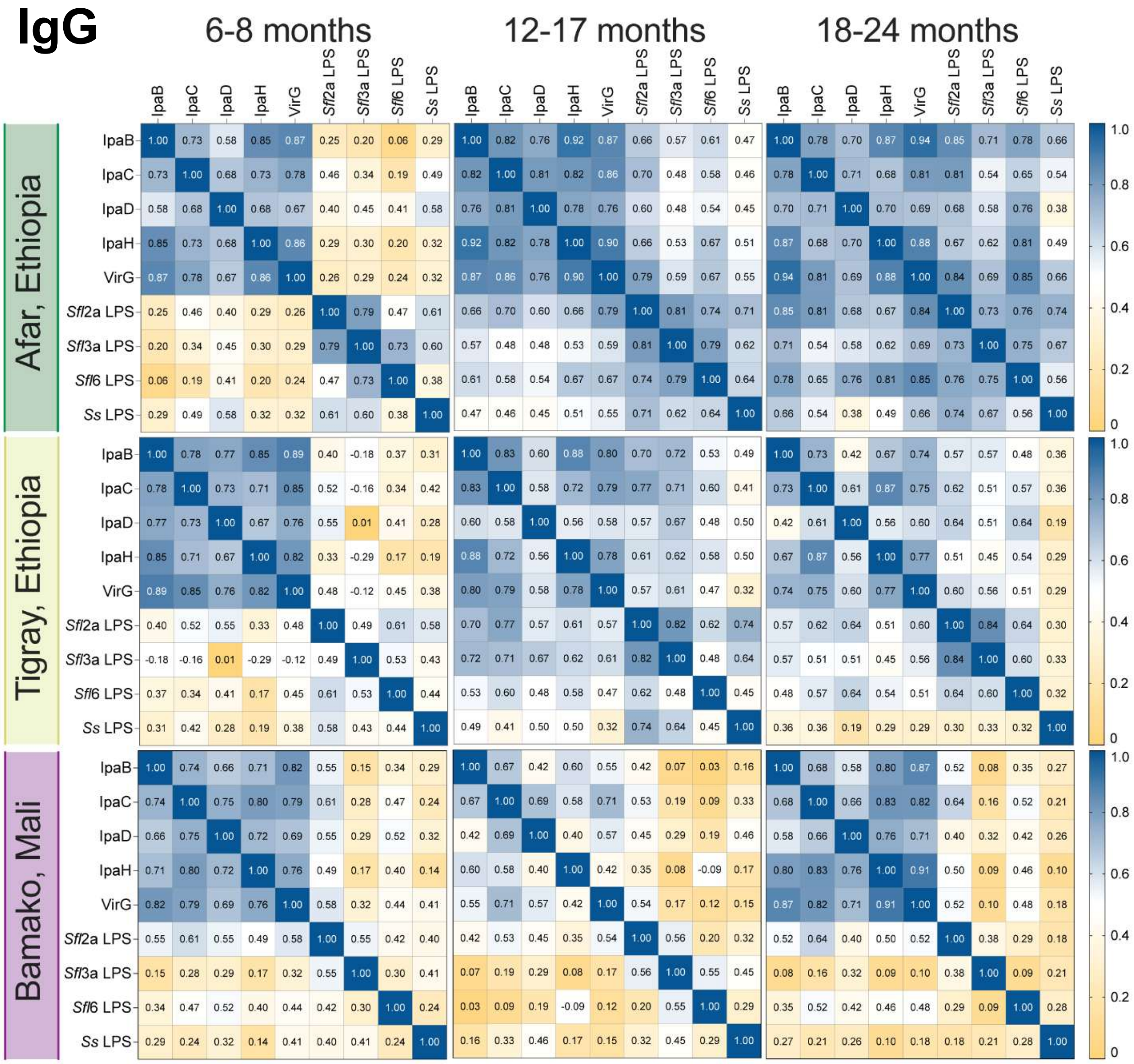

Figure S4. *Shigella* antigen-specific IgG correlations by age group and site. Heatmaps displaying correlations between antigen-specific IgG responses among children in Afar, Tigray, and Bamako. Spearman's correlation values are indicated in each cell.

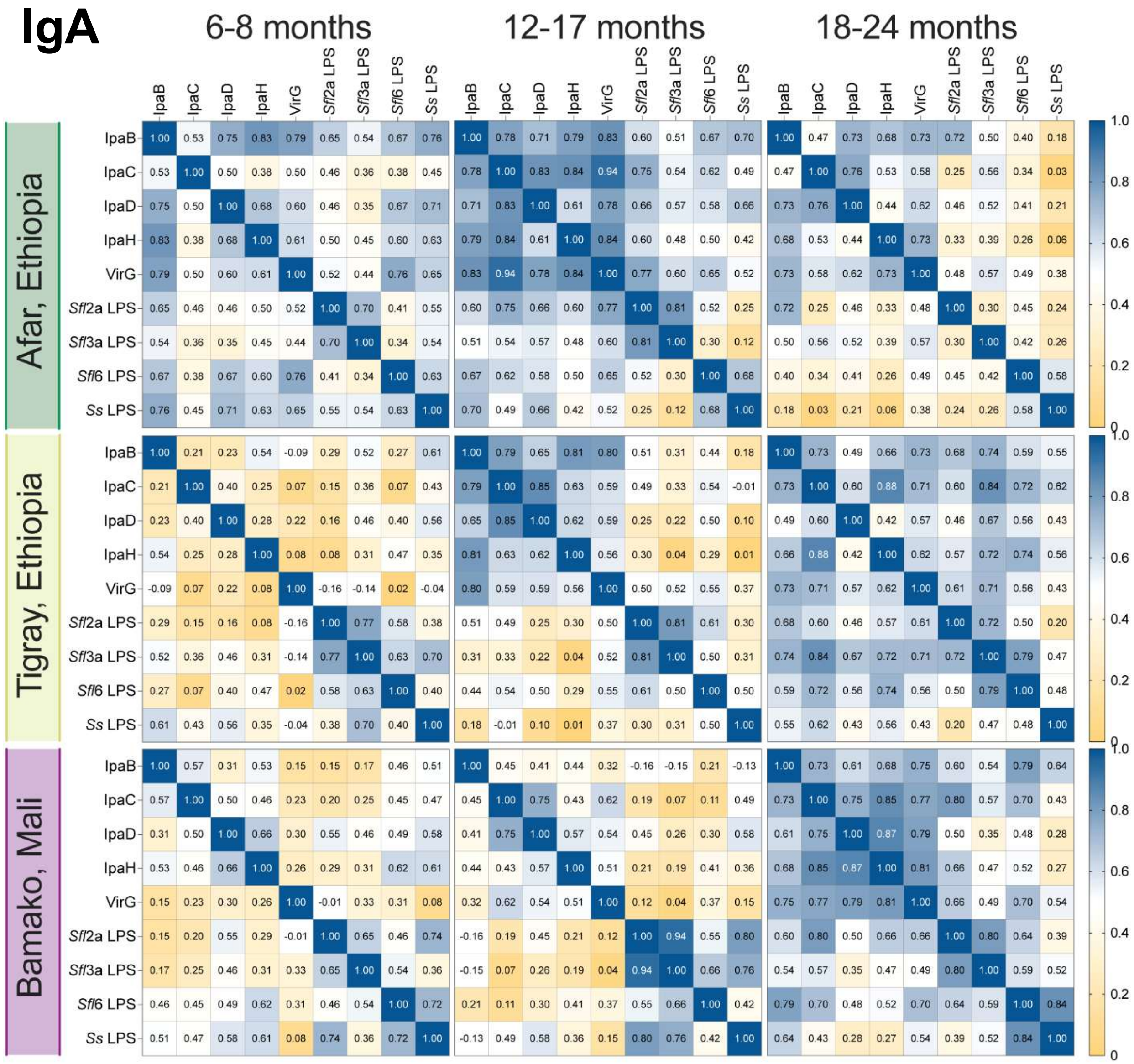

Figure S5. *Shigella* antigen-specific IgA correlations by age group and site. Heatmaps displaying correlations between antigen-specific IgA responses among children in Afar, Tigray, and Bamako. Spearman's correlation values are indicated in each cell.
